# Achievement of low-density lipoprotein cholesterol targets in Chinese patients with atherosclerotic cardiovascular disease after receiving statins and ezetimibe

**DOI:** 10.3389/fcvm.2022.988576

**Published:** 2022-10-14

**Authors:** Yinchu Cheng, Shujie Dong, Peng Shen, Yexiang Sun, Hongbo Lin, Suodi Zhai

**Affiliations:** ^1^Department of Pharmacy, Peking University Third Hospital, Beijing, China; ^2^Institute for Drug Evaluation, Peking University Health Science Center, Beijing, China; ^3^Yinzhou District Center for Disease Control and Prevention, Ningbo, China

**Keywords:** LDL-C, guideline, ASCVD, target achievement, real-world data

## Abstract

**Background:**

The importance of low-density lipoprotein cholesterol (LDL-C) lowering to reduce atherosclerotic cardiovascular disease (ASCVD) risk is strongly emphasized. If the LDL-C goals are not achieved with statin therapy, combination with ezetimibe is recommended. Studies revealed a substantial gap between obtained LDL-C levels and LDL-C target in ASCVD patients. However, little is known about the achievement of LDL-lowering treatment targets in ASCVD patients receiving ezetimibe in addition to statins.

**Materials and methods:**

This was a retrospective cohort study based on EHR data from the regional health information system of Yinzhou, an eastern coastal area of China. ASCVD Patients stratified as very high risk, taking both statin and ezetimibe for lipid control, and had at least one lipid test after ezetimibe initiation were included between January 2013 and July 2020. Descriptive statistics were used to summarize the LDL-C values and target value (1.8 mmol/L according to the Chinese guideline, 1.4 mmol/L according to the European guideline) achievements. Multivariable logistic regression was used to explore the influencing factors of target achievement rate.

**Results:**

A total of 1,727 patients were included. The median follow-up time was 15.0 months. Taking 1.8 mmol/L as the target value, the achievement rates of LDL-C over the first 3 follow up years were 50.6, 31.3, and 30.3%, respectively. Taking 1.4 mmol/L as the target value, the achievement rates were 25.6, 15.5, and 16.5%, respectively. Multivariable analysis suggested that male patients (OR = 1.78, 95%CI: 1.27–2.49), combined use of atorvastatin or rosuvastatin with ezetimibe (vs other statins, OR = 4.64, 95% CI: 1.83–11.76), better medication adherence (OR = 1.03, 95% CI: 1.01–1.04) and smoking cessation (vs smoking, OR = 2.26, 95% CI: 1.27–4.02) were associated with a higher achievement rate, while baseline LDL-C level (OR = 0.48, 95% CI: 0.41–0.56) and treatment course of statin before ezetimibe (OR = 0.93, 95% CI: 0.89–0.98) were negatively associated with achievement rate.

**Conclusion:**

Long-term follow-up data based on a Chinese regional database shows that in very high-risk ASCVD patients taking ezetimibe in addition to statins, achievement rate of LDL-lowering treatment targets is still low and far from satisfactory in real-world setting. More efforts are needed to achieve optimal LDL-C levels.

## Introduction

Atherosclerotic cardiovascular disease (ASCVD) is the leading cause of morbidity and mortality globally (1) and accounts for more than 40% of deaths in China (2). In previous guidelines on the management of dyslipidemia to reduce ASCVD risk in adults, the importance of low-density lipoprotein cholesterol (LDL-C) lowering to reduce ASCVD risk is strongly emphasized (3–5). Patients with documented ASCVD are considered to have very-high risk by the Chinese and European (4) guidelines, and LDL-C goals of < 1.8 mmol/L (<70 mg/dl) and < 1.4 mmol/L (<55 mg/dl) was recommended for these patients by the Chinese (5) and European guidelines (4), respectively.

Among lipid lowering drugs, statins are the cornerstone of therapy and are recommended to be prescribed up to the highest tolerated dose to reach the LDL-C goals set for the specific level of risk (3, 4). The addition of ezetimibe to statin therapy reduced LDL-C levels by an additional 21–27% compared with placebo (6). Moreover, it provided an additional benefit of lower risk of major vascular events to post acute coronary syndrome (ACS) patients (7). If the goals are not achieved with the maximum tolerated dose of a statin, combination with ezetimibe is recommended by guidelines (3–5).

Studies revealed a substantial gap between obtained LDL-C levels and LDL-C target in ASCVD patients. The Dyslipidemia International Study (DYSIS) showed that among 44,015 very-high-risk patients throughout 30 countries worldwide, only 21.7% attained their LDL-C goal of < 1.8 mmol/L (8). However, little is known about achievement of LDL-lowing treatment targets in ASCVD patients receiving ezetimibe in addition to statins. Using data from the Yinzhou Regional Health Care Database in China, we aimed to examine the target achievement rates in ASCVD patients after receiving statins and ezetimibe, and to explore the potential influencing factors of achieving LDL-C targets.

## Materials and methods

### Data source

Data of this study was extracted from the regional health information system of Yinzhou. Yinzhou is the largest district of Ningbo city, which locates in eastern China with nearly 1.5 million residents. The regional health information system was initiated by the local heath authority in 2005 to collect, store and manage the healthcare information of the city dwellers. It has covered all the hospitals and community health centers/stations in Yinzhou since 2010. Besides electronic health records (EHR) data, the system has also integrated public health data and several prospective disease registries managed by community general practitioners including cardiovascular disease, diabetes and cancer. In general, the health information system consisted of residents’ demographic characteristics, outpatient and inpatient hospital records (diagnosis, prescriptions, lab tests, etc.), death records and so forth. Details of this database has been described previously (9–12). For privacy protection, personal information and identifiers were anonymized. This study was approved by the ethical review board of Peking University Third Hospital (IRB00006761-M2019356), and informed consent of participant was exempted.

### Design and participants

This was a population-based retrospective cohort study based on the regional health information system of Yinzhou. Eligible patients meeting all the following criteria between January 2013 and July 2020 were included in the study: (1) patients who had at least one documented very high-risk ASCVD event/diagnosis, including coronary heart disease, acute coronary syndrome, myocardial infarction, ischemic stroke, transient ischemic attack, and peripheral arterial disease (identified using diagnosis/intervention text combined with ICD-10 codes, detailed in Supplementary Table 1); (2) patients who had at least 2 separate prescriptions of ezetimibe and, at the same time, had at least 1 prescription of statin both before and after the initiation of ezetimibe; (3) patients who had at least one record of lipid test after ezetimibe initiation. The index date was the date of the first ezetimibe prescription. Patients were followed up until the last date of record collection, death or the end of the study period (01 July 2020), whichever was earlier.

### Measurements

We evaluated patients’ lipid profiles 1, 2, and 3 years after ezetimibe initiation. In particular, the values of LDL-C were assessed. The target of LDL-C was set at below 1.8 mmol/L recommended by the Chinese guideline (5), as well as below 1.4 mmol/L according to the 2019 ESC/EAS Guidelines for the management of dyslipidemia (4).

Target achievement rate was defined as the number of patients who reached the target divided by the total number of patients. For patients who had multiple lipid tests, the lowest values of LDL-C during each follow-up year were chosen for the primary analyses, and the last value was used in the sensitivity analyses.

Patients’ baseline characteristics were measured at index date, including age, sex, BMI, lifestyle and the type of statin used. Baseline lipid profile was evaluated in the subgroup of patients who had lipid tests both before and after the index date. We also estimated the crude duration of ezetimibe and statin using the time interval between the first and last prescription of the relevant drug. Lipid lowering medication adherence was measured using the average number of patients’ statin or ezetimibe prescriptions per follow-up year as a proxy.

### Statistical analysis

Descriptive statistics were used to summarize patients’ demographic characteristics and lipid target achievement rates. Categorical variables were described using frequency counts and proportions. For continuous variables, normality was primarily assessed using the Kolmogorov–Smirnov test combined with histogram. If the data fitted normal distribution, means with standard deviations were calculated. Otherwise, medians with interquartile ranges (IQR) were used. The trend of patients initiating ezetimibe with time was also plotted.

Factors associated with LDL-C target achievement rate (measured using the lowest LDL-C value) during the whole follow up period were explored using multivariable logistic regression model. A bidirectional stepwise process was used to select variables to be included in the final model. Odds ratios (ORs) with 95% confidence intervals (CIs) were estimated.

As described above, alternative target and selection strategy for multiple lipid test results were applied in the sensitivity analyses. A two-sided P value less than 0.05 was considered strong evidence against the null hypothesis. All analyses were performed using the SAS software (version 9.4, SAS Institute Inc., Cary, NC, USA).

## Results

### Cohort selection, baseline characteristics and lipid treatments

**FIGURE 1:**
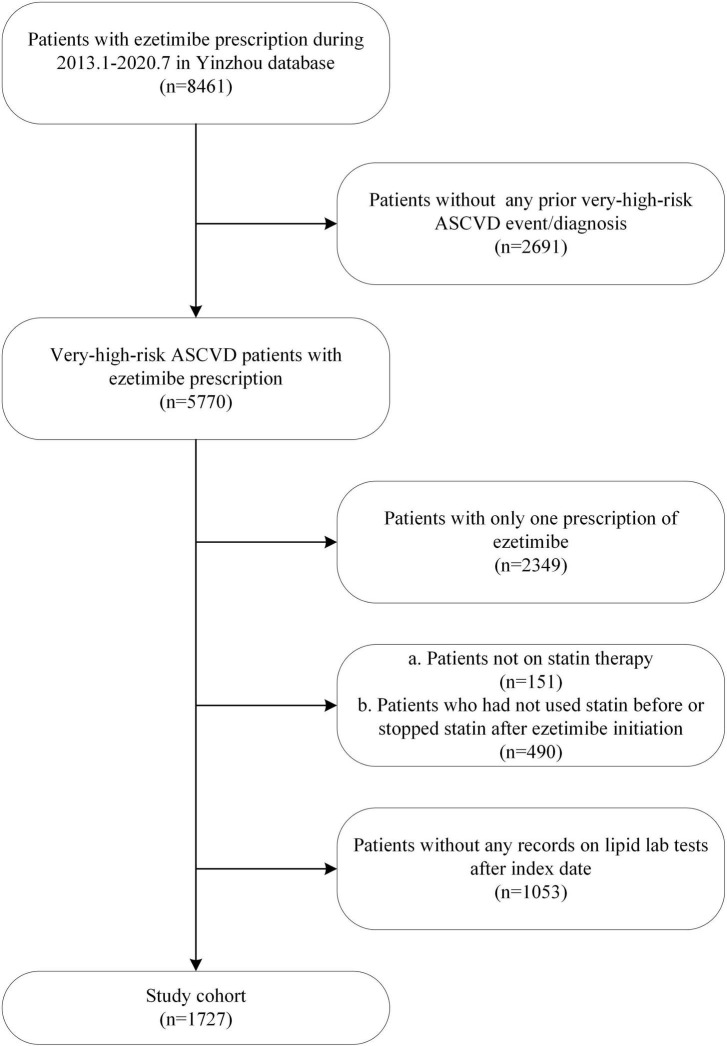
Flowchart of cohort selection.

Overall, 1,727 eligible patients were included in our study. The process of cohort selection is detailed in Figure 1. The median follow-up time was 15.0 months (IQR: 9.4-21.3). Among the patients with complete data at baseline, the median age was 64.0 years (IQR: 56.0-72.0), 79.37% were 45–74 years old, 48.87% were female, and 47.90% were overweight or obese. Behavioral habits including smoking status, alcohol consumption and exercise frequency are also described in Table 1. In terms of lipid status and treatment, patients’ median baseline LDL-C level was 2.91 (IQR: 2.14-3.80) mmol/L, and the most commonly used statins in combination with ezetimibe were atorvastatin and rosuvastatin (92.88%). The adherence of lipid lowering treatment, as measured by the number of patients’ statin or ezetimibe prescriptions per follow-up year, was 12.8 (IQR: 7.6-19.3). The median crude duration of treatment with statins and ezetimibe was 1,309 days (IQR: 680-2502) and 236 days (IQR: 124-439), respectively (Table 1).

**TABLE 1:** Baseline characteristics and lipid-lowering treatment of included patients.

Characteristics	Number of patients (*N* = 1,727)	Proportion (%)
**Sex (*n* = 1,723)**		
Male	881	51.13
Female	842	48.87
**Age-median (IQR), years (*n* = 1,726)**	64.0 (56.0–72.0)	
18∼44	72	4.17
45∼64	793	45.94
65∼74	577	33.43
≥75	284	16.45
**BMI (*n* = 1,359)**		
Low (<18.5)	35	2.58
Normal (18.5∼23.9)	673	49.52
Overweight (24.0∼27.9)	530	39.00
Obesity (≥28.0)	121	8.90
**Smoking status (*n* = 1,342)**		
Current smoker	197	14.68
Never	1,037	77.27
Ex-smoker	108	8.05
**Alcohol consumption (*n* = 1,341)**		
Never	1,046	78.00
Occasionally	10	0.75
Often	123	9.17
Everyday	162	12.08
**Exercise frequency (*n* = 1,335)**		
5–7 day/week	896	67.12
1–4 day/week	251	18.80
Seldom	188	14.08
**Baseline LDL-C-median (IQR), mmol/L (*n* = 1,541)**	2.91 (2.14–3.80)	
**Statin at ezetimibe initiation (*n* = 1,727)**		
Atorvastatin	1,103	63.87
Rosuvastatin	501	29.01
Simvastatin	112	6.49
Fluvastatin	10	0.58
Pitavastatin	1	0.06
**Number of statin/ezetimibe prescriptions per follow up year-median (IQR) (*n* = 1,727)**	12.8 (7.6–19.3)	
**Ezetimibe duration-median (IQR), days (*n* = 1,727)**	236.0 (124.0–439.0)	
**Statin duration-median (IQR), days (*n* = 1,727)**	1,309.0 (680.0–2502.0)	

**FIGURE 2:**
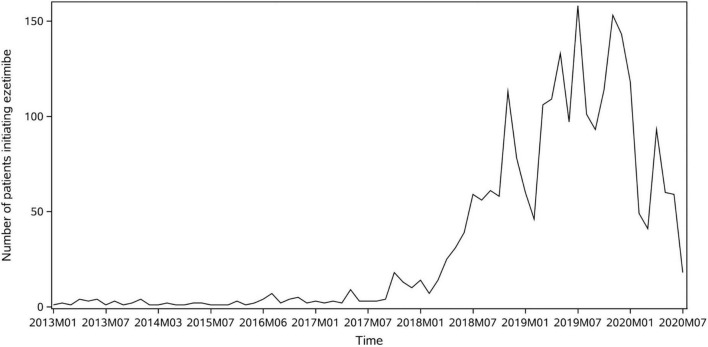
Number of patients starting to use ezetimibe over time in Yinzhou database.

The background utilization trend of ezetimibe in Yinzhou Database is shown in Figure 2 by plotting the number of ezetimibe new users over time, which indicates a major increase in ezetimibe utilization since year 2018. The sharp dips at the beginning of 2020 might be correlated with the outbreak of COVID-19.

### Lipid profile and target achievement

**FIGURE 3:**
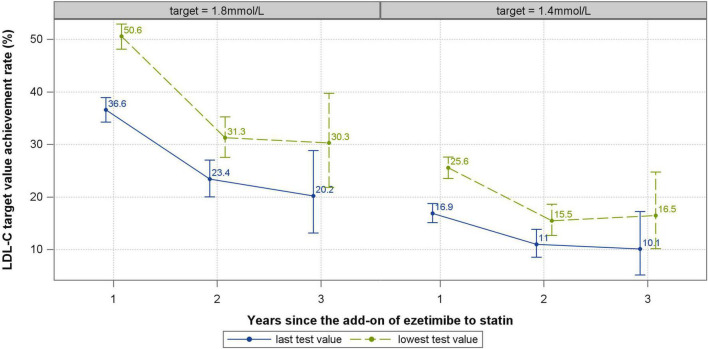
LDL-C target value achievement rate first 3 years after ezetimibe plus statin. The vertical lines and whiskers indicate the 95% confidence intervals of the relevant rates.

LDL-C values and target value achievement rates over 1–3 years after statin plus ezetimibe are shown in Table 2 and Figure 3. The overall number of patients with available lipid tests decreased rapidly with time. Based on the primary analyses using the lowest lipid test values during each follow up year, the median LDL-C values 1–3 years after ezetimibe initiation were 1.79 (IQR: 1.41-2.43), 2.23 (IQR: 1.66-2.79) and 2.19 (IQR: 1.72-2.98) mmol/L, respectively. Taking 1.8mmol/L as the target value, the achievement rates of LDL-C over the first 3 follow up years were 50.6, 31.3, and 30.3%, respectively, which were far from satisfactory and declined over time. Taking 1.4 mmol/L as the LDL-C target value, the achievement rates over the years were 25.6, 15.5, and 16.5%, respectively, which were generally low and declined significantly after the first year. Sensitivity analyses results based on the last lipid test values during each follow up year showed higher LDL-C values and lower achievement rates after statin plus ezetimibe (Table 2).

**TABLE 2:** Lipid values and target achievement rates by years since statin plus ezetimibe.

LDL-C values and targets	LDL-C values (median (IQR), mmol/L) and target value achieve rates (n of achiever, %) by years after ezetimibe initiation		
	Year 1 (*n* = 1,643)	Year 2 (*n* = 556)	Year 3 (*n* = 109)
**Lowest test value**	1.79 (1.41–2.43)	2.23 (1.66–2.79)	2.19 (1.72–2.98)
<1.8 mmol/L	831 (50.6%)	174 (31.3%)	33 (30.3%)
<1.4 mmol/L	420 (25.6%)	86 (15.5%)	18 (16.5%)
**Last test value**	2.10 (1.58–2.85)	2.42 (1.86–3.07)	2.48 (1.94–3.23)
<1.8 mmol/L	601 (36.6%)	130 (23.4%)	22 (20.2%)
<1.4 mmol/L	277 (16.9%)	61 (11.0%)	11 (10.1%)

### Factors associated with low-density lipoprotein cholesterol target achievement rate

**FIGURE 4:**
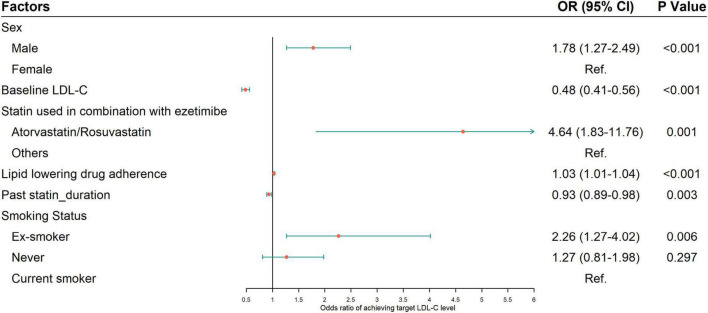
Factors associated with LDL-C target value achievement rate from multivariable logistic regression. Variables in the original model included age, sex, statins used in combination with ezetimibe, baseline LDL-C, body mass index, smoking status, alcohol consumption, exercise habits, lipid lowering drug adherence and treatment courses of statin and ezetimibe. A bidirectional stepwise process was applied to select variables to be included in the final model. OR, odds ratio.

Multivariable logistic regression was used to explore the factors associated with LDL-C target achievement rate, and the results are presented in Figure 4. The data suggested that male patients (vs female, OR = 1.78, 95%CI: 1.27–2.49), combined use of either atorvastatin or rosuvastatin with ezetimibe (vs other statins, OR = 4.64, 95% CI: 1.83–11.76), better medication adherence (every incremental prescription of statin/ezetimibe per year, OR = 1.03, 95% CI: 1.01–1.04) and smoking cessation (vs smoking, OR = 2.26, 95% CI: 1.27–4.02) were associated with a higher achievement rate. Higher baseline LDL-C level (per mmol/L increase, OR = 0.48, 95% CI: 0.41–0.56), and longer treatment course of statin before ezetimibe (per year increase, OR = 0.93, 95% CI: 0.89–0.98) were associated with a lower achievement rate.

## Discussion

In this retrospective cohort using the Yinzhou Regional Health Care Database in China, we examined the target achievement of LDL-C levels in ASCVD patients on statins and ezetimibe combination treatment. Only 50.6 and 25.6% of ASCVD patients met the Chinese and European guideline recommendations in the first year, respectively. Of note, the achievement rates and LDL-C levels decreased after the first year. Unsatisfactory LDL-C target achievement rates have been consistently reported in previous studies, however, most of them were focusing patients on statin monotherapy. In the Chinese cohort of DYSIS, 23.9% of patients with very-high risk achieved the LDL-C goal (<1.8 mmol/L) between the year 2006 and 2013 (13). Our study only focused on patients receiving both statins and ezetimibe who has so far received limited attention, and the achievement rate didn’t seem to improve.

Combined statin and ezetimibe therapy are underused in patients with ASCVD in real-world practice. In the DYSIS study, only 6.8% patients were treated with a statin plus ezetimibe (8). In a Japanese study of 33,000 high risk patients, 24% patients on high-intensity statin were concomitantly prescribed with ezetimibe, and only 5% patients on low- to moderate-intensity were concomitantly prescribed with ezetimibe (14). In an analysis of a real practice database in Italy, 71.1% patients were prescribed with statin and only 3.3% patients were prescribed with ezetimibe for secondary prevention (15). It is unexpected that even though the patients were on combined therapy, the rate of target achievement of LDL-C levels in ASCVD patients is still far from satisfactory in our study. The proportions of patients with LDL-C < 1.8 mmol/L and < 1.4 mmol/L were reported to be 38.5 and 17.5% among those on statin and ezetimibe treatment in Italy (16). Our study showed even lower LDL-C achievement rates in year 2 and year 3 than those in Italy. Since our primary analysis is based on the lowest values of multiple lipids tests, the actual achievement rates might be even worse.

The most important way to prevent ASCVD is to promote a healthy lifestyle throughout life (1). Smoking is a definite cardiovascular risk factor (1). In our analysis, smoking cessation was found to be a positive influencing factors of achieving LDL-C targets, indicating a further lifestyle optimization encouragement of smoking cessation in ASCVD patients. Longer treatment course of statin before ezetimibe was identified as a risk factor for a lower achievement rate. Therapeutic inertia, which refers to failure to intensify therapy when treatment targets are not met, can occur at the level of both physicians and patients (17). It may be a possible explanation for delayed non-statin therapy addition when LDC-C targets were not met even after a long treatment course. Efforts to early initiate ezetimibe is essential for ideal achievement rates.

Given the unsatisfactory attainment, further intensive efforts are needed to achieve optimal LDL-C levels. Our study highlight the need for physicians to improve guideline adherence. Previous studies identified barriers as awareness, familiarity, agreement, self-efficacy, outcome expectancy, ability to overcome the inertia of previous practice, etc. (18). Physician attitude on the ideal goal for LDL-C level was an important factor associated with intensified therapy and LDL-C level achievement (19). Appropriate interventions, taking important factors for barriers in account, are necessary to improve guideline implementation. In addition, poor medication adherence, commonly seen in patients taking lipid-lowering drugs (20–22), has been found by our study and also previous studies to be one of the most important modifiable factors that compromises treatment outcomes (23). Using a fixed combination of ezetimibe/statin therapy, improving patient awareness, increasing availability of medical support and self-management support can help promote patient adherence (23).

Monoclonal antibodies that inhibit proprotein convertase subtilisin/kexin type 9 (PCSK9) have been shown to lower LDL-C levels on average by 60% and provide a further reduction in ASCVD risk (24–27). Therefore, combined use of PCSK9 inhibitors should be taken into consideration for patients at very-high risk not achieving their goals on a maximum tolerated dose of a statin and ezetimibe. None of the patients in our study were prescribed PCSK9 inhibitors, since PCSK9 inhibitors just came into Chinese market then and were not covered by medical insurance. Additionally, two new drugs, bempedoic acid and inclisiran, have recently been approved and provided potential choices for the lowering of LDL-C levels (28). Cost has been a hurdle for use of new therapies, and recent price reductions may trigger increased use.

It is important to note that this study may have certain limitations. Firstly, our population was limited to residents of Yinzhou, an eastern coastal area of China with economic level above average. Thus, the sample size of our study is relatively small compared with other studies (6, 8, 14) and caution should be warranted when extrapolating our findings to other populations. Secondly, low frequency of lipid tests is observed in this analysis, which limited the sample size to estimate the achievement rates, thus reducing the precision and robustness of the study results. Despite this, given that the database has covered all the hospitals and community health centers/stations in Yinzhou, the study data provide the best-available picture of this regional lipids achievement over 3 years. Thirdly, most of ezetimibe prescriptions occurred after 2018, leading to a relatively short duration of follow up. Future studies are needed to evaluate patients’ lipid profiles over longer time periods. Finally, records on statin doses were insufficient in the database, making it difficult to further discuss different combination with high-, moderate-, low-intensity statin therapy. The incompleteness of dosage information also resulted in the inability to evaluate patients’ medication adherence directly with the commonly used index of medication possession rate. To address this issue, we used the average number of patients’ statin or ezetimibe prescriptions per follow-up year as a proxy of adherence. The results turned out to be consistent with previous studies in revealing the importance of medication adherence in lipid lowering treatment outcome (20–22).

## Conclusion

Long-term follow-up data based on a Chinese regional population shows that in very high-risk ASCVD patients taking ezetimibe in addition to statins, achievement rate of LDL-lowering treatment targets is still low and far from satisfactory in real-world setting. More efforts are needed to achieve optimal LDL-C levels recommended by guidelines.

## Data availability statement

The original contributions presented in this study are included in the article/Supplementary material. Requests to access these datasets should be directed to HL, lin673160@163.com.

## Ethics statement

The studies involving human participants were reviewed and approved by Peking University Third Hospital, China. Written informed consent for participation was not required for this study in accordance with the national legislation and the institutional requirements.

## Author contributions

YC, SD, and SZ conceived the idea for this manuscript. HL, PS, and YS extracted the data from the database. YC conducted the statistical analysis. YC, SD, HL, and SZ contributed to the interpretation of the results. YC and SD wrote the manuscript. HL, PS, YS, and SD contributed to the revision of the manuscript. All authors agreed to be accountable for the content of the work.
