# Supplementary material for: Achievement of low-density lipoprotein cholesterol targets in Chinese patients with atherosclerotic cardiovascular disease after receiving statins and ezetimibe
Source: Front Cardiovasc Med. 2022 Oct 14;9:988576. doi: 10.3389/fcvm.2022.988576 (PMC9614052; doi:10.3389/fcvm.2022.988576)
Supplement: Supplementary file 1 [file Table_1.DOCX]

**Supplementary**

**Table 1 ASCVD diagnosis/ interventions and ICD codes**

| **ASCVD diagnosis/ Interventions** | **ICD-10** |
| --- | --- |
| Angina  Atherosclerotic cardiovascular disease  Atherosclerotic heart disease | I20, I25.0, I25.1 |
| Acute coronary syndrome  Acute myocardial ischemia | I24.8, I24.9 |
| Myocardial infarction | I21, I22, I23, I25.2 |
| Cerebral infarction/Ischemic stroke | I63 |
| Transient ischemic attack | G45.4, G45.9 |
| Peripheral arterial disease | I65.2, I65.0, I70.1, I70.2, I70.8, I74.2, I74.3, I74.4, I74.5, I74.8, I77.107, I77.106, I77.109, N28.0, I73.1, I73.902, I73.904 |
| Percutaneous coronary intervention (PCI) | / |
| Coronary artery bypass grafting (CABG) | / |
